# Genomic comparisons confirm *Giardia duodenalis* sub-assemblage AII as a unique species

**DOI:** 10.3389/fcimb.2022.1010244

**Published:** 2022-10-17

**Authors:** Matthew H. Seabolt, Dawn M. Roellig, Konstantinos T. Konstantinidis

**Affiliations:** ^1^ Division of Foodborne, Waterborne, and Environmental Diseases, National Center for Emerging and Zoonotic Infectious Diseases, Centers for Disease Control and Prevention, Atlanta, GA, United States; ^2^ School of Biological Sciences, Georgia Institute of Technology, Atlanta, GA, United States; ^3^ Public Health Office, Leidos Inc., Reston, VA, United States; ^4^ School of Civil and Environmental Engineering, Georgia Institute of Technology, Atlanta, GA, United States

**Keywords:** *Giardia*, assemblage A, comparative genomics, cryptic species, phylogenetics, zoonotic diseases

## Abstract

*Giardia duodenalis* is a parasitic flagellated protozoan which infects a wide range of mammalian hosts, including humans, and is subdivided into at least eight genetic assemblages commonly thought to represent cryptic species. Molecular studies have shown that *G. duodenalis* assemblage A, which parasitizes humans and animals, contains several phylogenetically distinct groupings known as sub-assemblages. Molecular studies employing poor phylogenetic-resolution markers routinely recover these sub-assemblages, implying that they represent evolutionarily distinct clades and possibly cryptic species, a hypothesis which is supported by epidemiologic trends. Here, we further tested this hypothesis by using available data from 41 whole genomes to characterize sub-assemblages and coalescent techniques for statistical estimation of species boundaries coupled to functional gene content analysis, thereby assessing the stability and distinctiveness of clades. Our analysis revealed two new sub-assemblage clades as well as novel signatures of gene content geared toward differential host adaptation and population structuring *via* vertical inheritance rather than recombination or panmixia. We formally propose sub-assemblage AII as a new species, *Giardia hominis*, while preserving the name *Giardia duodenalis* for sub-assemblage AI. Additionally, our bioinformatic methods broadly address the challenges of identifying cryptic microbial species to advance our understanding of emerging disease epidemiology, which should be broadly applicable to other lower eukaryotic taxa of interest. Giardia hominis n. sp. Zoobank LSID: urn:lsid: zoobank.org:pub:4298F3E1-E3EF-4977-B9DD-5CC59378C80E.

## Introduction

*Giardia duodenalis* (syn. *G. intestinalis*, *G. lamblia*) is a common intestinal protozoan parasite of humans, domestic animals and livestock, and wildlife. *Giardia* infection is acquired by ingestion of contaminated water or food, or by the fecal-oral route for person-to-person contact and is typically characterized by self-limiting illness (giardiasis) but can cause long-term symptoms like irritable bowel syndrome and chronic diarrhea. Giardiasis has also been linked to long-term effects in children such as stunting, malnutrition, and impaired development. Giardiasis is a major global public health concern, having the potential to cause large waterborne outbreaks, with 1.2 million estimated cases occurring in the United States and upwards of 280 million cases worldwide each year (Painter et al., 2015; Einarsson et al., 2016; Coffey et al., 2021). In the United States, hospitalizations from giardiasis cost an estimated $34 million annually (Painter et al., 2015). Clinical symptoms include diarrhea, bloating, abdominal cramps, malabsorption, dehydration, nausea, vomiting, and weight loss (Painter et al., 2015; Eckmann, 2003; Cacciò and Ryan, 2008). Asymptomatic infections are also commonly reported, especially in developing countries (Painter et al., 2015). *G. duodenalis* can be divided into eight genetic groups, called assemblages, named A through H, which are morphologically indistinguishable Assemblages A and B have broad host ranges that include humans, and thus are considered zoonotic pathogens, while the remaining assemblages (C-H) are typically host-adapted and pose low infection risk to humans (Feng and Xiao, 2011; Thompson and Ash, 2016; Cacciò et al., 2008).

*G. duodenalis* is now commonly regarded as a multispecies complex and proposals have been put forth to elevate the assemblages to the rank of species based on allozyme, molecular, and natural history data (Cacciò and Ryan, 2008; Monis et al., 1999; Sprong et al., 2009; Thompson and Monis, 2011). Comparisons between available draft genomes confirm that the genetic distances separating assemblages are substantial and consistent with recognizing each assemblage as a separate species (Franzen et al., 2009; Jerlström-Hultqvist et al., 2010; Adam et al., 2013; Wielinga et al., 2015; Seabolt et al., 2021). However, taxonomic revisions have not yet materialized due to failure to reach consensus about historical nomenclature and uncertainty about historical isolate characterization (Thompson and Monis, 2011; Ryan and Cacciò , 2013). Further, genetic structure within assemblages is recognized based on allozymes and DNA sequencing of several gene targets, namely the SSU-rDNA, glutamate dehydrogenase (*gdh)*, beta-giardin (*bg*), and triosephosphate isomerase (*tpi*) genes, which allows isolates to be classified into sub-assemblages, albeit with mixed concordance between methods (Ryan and Cacciò, 2013; Cacciò et al., 2008).

Accurate classification of *G. duodenalis* isolates derived from human infections is important for public health due to the zoonotic potential of assemblages A and B, and improved classification schemes might help inform outbreak investigations, source tracking, understanding of transmission dynamics, and prevention strategies. In this work, we focus on Assemblage A, which has a global distribution and is responsible for 37% of human infections annually (an estimated 75 million cases; Feng and Xiao, 2011). Assemblage A can be reliably differentiated into three stable phylogenetic clusters (sub-assemblages AI, AII, and AIII), which differ in host preference (Cacciò et al., 2008; Sprong et al., 2009; Tsui et al., 2018). Human infections are most commonly identified as sub-assemblage AII while animals are more commonly infected with sub-assemblage AI, although AI has been occasionally identified in human cases, and vice versa with animal infections caused by AII (Xiao and Fayer, 2008; Sprong et al., 2009; Ankarklev et al., 2018). Sub-assemblage AIII has been found almost exclusively in wild ruminants (Robertson et al., 2007; Solarczyk et al., 2012). Key genetic differences between sub-assemblages correlating with host preference or epidemiology have yet to be robustly identified using existing typing methods. These sub-assemblage units have been thus far absent from the discussion of elevating *G. duodenalis* assemblages to the species rank, despite being distinguishable by the same original criteria that are cited as justification for species recognition of the other assemblages. Thus, these assemblage A subgroups may also be deserving of species rank (Cacciò et al., 2008; Thompson and Monis, 2011; Cacciò et al., 2008; Tsui et al., 2018). Recognition of species boundaries, traditionally *via* reproductive isolation (RI) or the Biological Species Concept, is challenging in *Giardia* due to the paucity of morphological characters and poor understanding of the mechanisms that generate novel genetic diversity (e.g. sexual recombination, horizontal gene transfer), necessitating statistical methods to discriminate between diverse populations and isolated cryptic species. Our aims in this work were to quantify population structure and relationships between sub-assemblage units using available whole-genome sequences of *G. duodenalis* assemblage A, and to evaluate whether these relationships constitute sufficient evidence supporting elevation of sub-assemblage clades to the species level under the same criteria as other *G. duodenalis* assemblages. We synthesize data on genomic relatedness, gene content diversity, and population genetics as a proxy for RI to support our conclusions for statistical species delimitation in *Giardia*.

## Materials and methods

### Sampling, data pre-processing, and pangenome construction

Available whole-genome sequencing reads and assemblies from 41 isolates of *Giardia duodenalis* assemblage A were downloaded from the Sequence Read Archive (SRA) and Assembly databases at NCBI in January 2020 (**Table S1**). The sequenced *Giardia* strains were originally collected as part of monitoring projects and outbreak investigations between 1989 and 1995 or are available through ATCC (American Type Culture Collection); one genome was derived from a human patient circa 2015 (Morrison et al., 2007; Adam et al., 2013; Prystajecky et al., 2015; Ankarklev et al., 2015; Tsui et al., 2018; Xu et al., 2020; ). Reads from each SRA accession were quality assessed using fastQC and subsequently trimmed using bbduk to remove low quality bases less than Phred score 20, Illumina adapter sequences, and reads shorter than 50 nt in length (Bushnell, 2014). Quality controlled reads were assembled by mapping against the “WB” isolate reference genome using bbmap (Bushnell, 2014). Unmapped reads were extracted from the SAM file and *de novo* assembled using idba-ud (Peng et al., 2012). *De novo*-assembled contigs less than 500 nt in length were filtered out, and the remaining contigs were added to the assembly for each genome. Pangenome genes were predicted for all genomes using the program augustus and a previously trained HMM model for *Giardia duodenalis*, which we re-optimized using the updated WB reference annotations (Hoff and Stanke, 2019; Xu et al., 2020; Seabolt et al., 2021). Predicted gene sequences were pooled and clustered using the usearch –cluster_fast algorithm with parameters of 90% nucleotide identity across 90% of the sequence length required for inclusion in each cluster (Edgar, 2010). The sequence which was best representative of each cluster was exported to form the final set of pangenome genes. Finally, to identify orthologs, each gene in the pangenome set was searched against each genome assembly with blastn (Altschul et al., 1997), extracting the reciprocal best match when it showed a minimum of 70% nucleotide identity covering at least 70% of the query sequence. Blast results were further parsed to generate a phyletic gene content matrix. Functional annotation terms were assigned to each gene by protein homology searches against the eggNOG database with an e-value threshold of 1e-6 (Huerta-Cepas et al., 2016). Identifiers and annotations from the NCBI reference files were additionally retained for genes that showed reciprocal best matches to a previously annotated locus in the WB genome. Genes were categorized as core pangenome if a gene appeared in all genomes, unique if the gene appeared in only one genome, and as an accessory gene if it appeared in at least two genomes but not all. Sub-assemblage assignment was determined for each genome using the multilocus genotyping (MLG) scheme described in Cacciò et al. (2008) and Sprong et al. (2009), which utilizes the triose phosphate isomerase (*tpi*), glutamate dehydrogenase (*gdh*), and beta-giardin (*bg*) genes. Under this typing scheme, two genomes (SRR3177751 and SRR3177919) each represented a distinct genotype that was assignable to neither sub-assemblage AI, AII, nor AIII, and were thus designated as Ax and Ay, respectively, for the purposes of downstream comparisons.

### Phylogenetic analyses and statistical species delimitation

Nucleotide alignments were built for all individual genes in the pangenome using mafft v7 (Katoh and Standley 2013). The best-fitting evolutionary model was estimated using jModelTest2 based on the Akaike information criteria (AIC) (Darriba et al., 2012). Maximum likelihood (ML) gene trees were computed using phyml v20120412 using the selected substitution model plus the best starting tree from jModelTest2 as input (Guindon and Gascuel, 2003) and setting the random seed value to 7. We also estimated a neighbor-joining (NJ) tree using distances calculated from pairwise average nucleotide identity (ANI) comparisons of draft genomes using the software fastANI (Jain et al., 2018). Clonal complex assignments were determined using maximal clique enumeration (MCE, a graph-based clustering method) with a cutoff of 99.9% ANI and provisionally named in sequential order following Seabolt et al. (2021). To test for putative boundaries between cryptic species, we utilized two multilocus coalescent-based approaches (stacey v1.2.4 and astral-III v5.6.3). The set of all ML gene trees was used as input to astral-III, which estimates the species tree based on unrooted shared quartets in the set of gene trees (Zhang et al., 2018) and was run with default parameters with the random starting seed set to 14. stacey is a plugin for the beast2 package, which uses a Bayesian likelihood algorithm to estimate the species tree (Bouckaert et al., 2014; Jones, 2017). To construct the evolutionary model file for stacey, we filtered out loci with missing genomes in the alignment and ≥ 1500 nt in length, retaining 2142 loci after filtering. We specified a maximum length cutoff of 1500 nt because longer loci are more likely to be captured by recombination (Singhal et al., 2018). Each locus was configured to have an uncorrelated relaxed lognormal clock, GTR substitution model with empirical rate frequencies, and Yule Process priors. Specific priors were set as follows: growthRate ~ lognormal(µ=5, σ=2), collapseHeight = 1e-4, collapseWeight ~ Uniform(0,1), populationPriorScale ~ lognormal(µ=-5, σ=2), and relativeDeathRate ~ β(α=1.0, β=5.0). We then ran stacey for 10 million generations, sampling trees every 1000 generations. Statistical species delimitation was computed by speciesDA v1.8.0 (Jones 2015) using the sampled trees from stacey, a burn-in fraction of 10%, and collapse height parameter of 1e-4. The robustness of the results was confirmed by repeating the analysis with collapse heights of 1e-3 and 1e-5. The resulting phylogenies from astral, stacey, and fastANI were visualized and annotated using the Interactive Tree of Life (iTOL) web portal (Letunic and Bork, 2019; URL: https://itol.embl.de).

### Functional gene content comparisons

In addition to phylogenetic delimitation of putative cryptic species, we also sought to identify biological differences between sub-assemblages based on gene content differences captured in the pangenome. First, to estimate the “openness” of the pangenome, we calculated a rarefaction curve and the Heaps law parameter α using the R package *micropan* and 100 permutations in both operations (Snipen and Liland 2019). We next analyzed the evolutionary history of gene gain and loss using the stochastic GainLoss Mapping Engine (gloome) with the phyletic matrix and the ANI reference phylogeny as inputs (Cohen et al., 2010). gloome estimates probabilities of gain or loss (i.e. presence or absence) of genes along each branch in the reference phylogeny using the observed phyletic data and a continuous Markov model, which allows variable evolutionary rates per branch. Only gain/loss events with probability greater than 0.95 were retained for each lineage. We further investigated gene content differences between lineages by assigning the gene complement of each genome to a collective gene pool per sub-assemblage, clonal complex, and host, followed by group-wise comparisons using mathematical Set operations and custom Perl scripts. Gene pools for each sub-assemblage were compared against the total pangenome and pairwise to one another to identify genes unique to one sub-assemblage as well as genes absent from a given gene pool using custom Perl scripts. Finally, the phyletic matrix was used to estimate a tree using the dist.binary(method = 5) function and NJ algorithm implemented in the R packages *ade4* and *ape* (Dray and Dufour, 2007; Paradis et al., 2004) with 1000 bootstrap replicates.

### Variant detection and population genetic analyses

The representative (centroid) sequence of each core gene cluster was chosen to construct a pseudo-reference genome. Single nucleotide polymorphism (SNP) data were generated for each individual sample by mapping reads to the pseudo-reference genome with bbmap and calling variant sites using gatk v3.8 HaplotypeCaller with a workflow adapted from the gatk v3.8 Best Practices for germline short variant detection (McKenna et al., 2010). Variants with quality ≤ 20, read depth (DP) ≤ 10x, and quality depth (QD) ≤ 5 were filtered out following GATK’s recommended filters. All variant calls passing filter were pooled into a combined VCF file per lineage (=population). The program snpEff (Cingolani et al., 2012) was used to annotate variant calls and filter for variants annotated as synonymous SNVs.

The goals of our population genetic analyses were to describe patterns of genetic diversity and divergence among the putative cryptic lineages. To this end, we first evaluated potential population structure within and among genomes using Wright’s *F _st_* (Wright, 1943; Weir and Cockerham, 1984) and a three-level hierarchical analysis of molecular variance (AMOVA; Excoffier et al., 1992). All population genetic analyses were computed using synonymous (silent) sites only. In brief, *F _st_* ranges from 0 to 1, with a value of 0 suggestive of complete panmixis (no population structuring) and a value of 1 suggestive that all variation at a given locus infers population structuring, conditional on barriers to gene flow between the populations being compared. Pairwise *F _st_* between each lineage were calculated with the R package *stAMPP* (Pembleton et al., 2013), assessing significance with 1000 bootstrap replicates. Per-site *F _st_* values calculated using all genomes and 1000 bootstrap replicates were averaged to estimate the genome-wide *F _st_ *. AMOVA calculations were performed using the *poppr* package in R (Kamvar et al., 2014), again assessing significance by bootstrapping (replicates=1000), and three hierarchical levels: (i) the assigned assemblage, (ii) the genome’s continent of origin, and (iii) among genomes within an assemblage. To further characterize patterns among the genomes, we computed within-lineage and pairwise estimates of genetic diversity (π; Nei and Li, 1979), raw and net divergence (D _xy_ and D_a_ respectively; Nei and Li, 1979), and two tests for neutral evolution (Tajima’s D, Tajima, 1989a; Fu and Li’s D* and F*, Fu and Li, 1993) using the program DNASP v5 (Librado and Rozas, 2009).

## Results

### Statistical support for cryptic species

**Figure 1:**
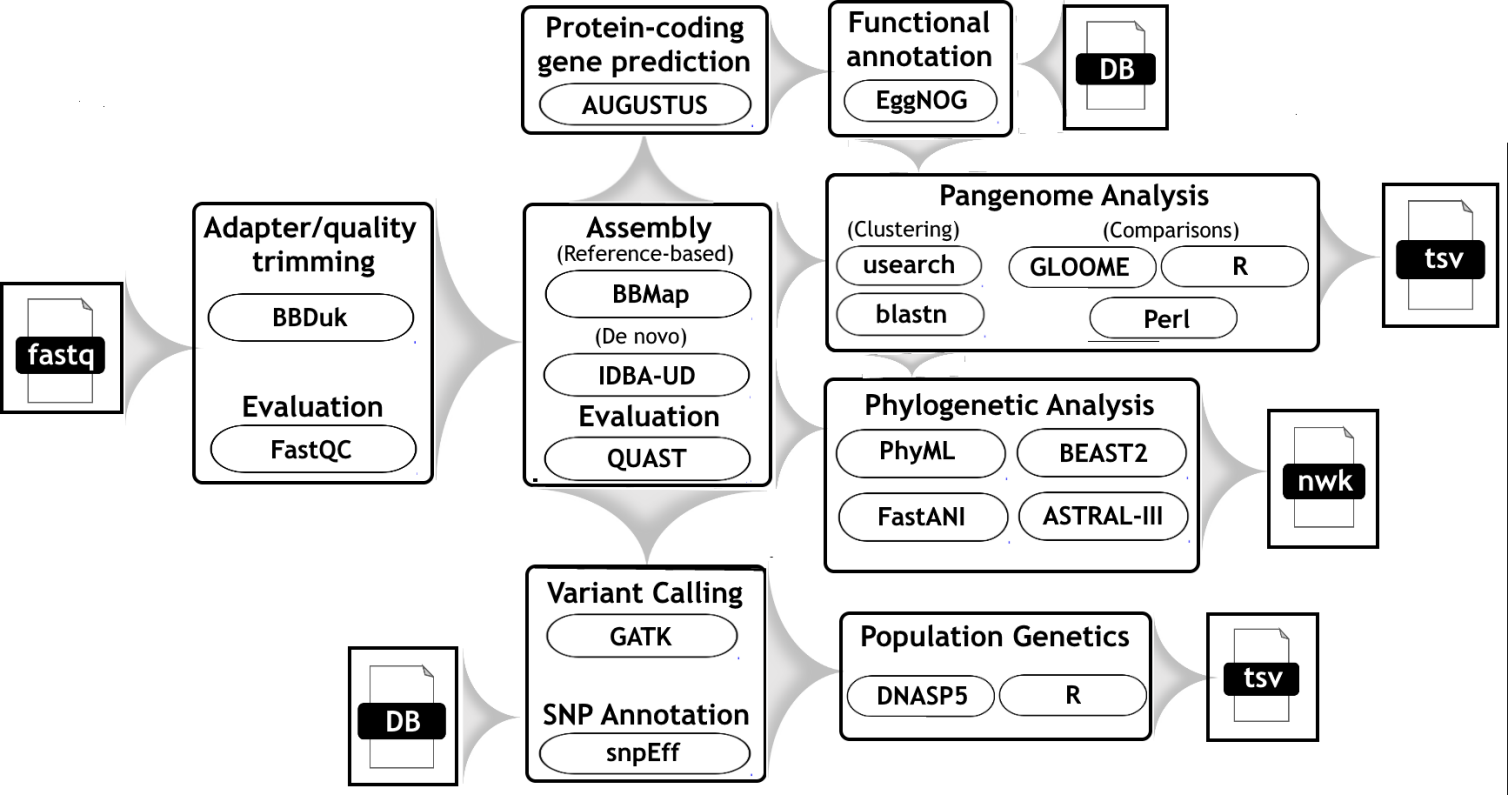
Workflow diagram which describes the bioinformatic steps taken to differentiate genomes of *G. duodenalis* assemblage A strains.

The set of genomes included in our study represented sub-assemblages AI (n=29) and AII (n=10), and two genomes each representing novel lineages that were not assignable to any recognized sub-assemblage and were provisionally denoted as Ax and Ay. The genome sequences represented (i) sampling locations in North America (Canada and USA; n=36), Europe (Sweden and UK; n=2), and New Zealand (n=3), three of which were associated with travel to Afghanistan (WB, sub-assemblage AI), Mexico (SRR3177750, sub-assemblage AII), and Kenya/Sudan (SRR3177990, sub-assemblage AII), and (ii) human, beaver, sheep, cat, dog, and environmental (surface water) isolates. Additional sample metadata is detailed in **Table S1**. The bioinformatics workflow used to analyze these genomes is described in detail below and summarized in **Figure 1**.

**Figure 2:**
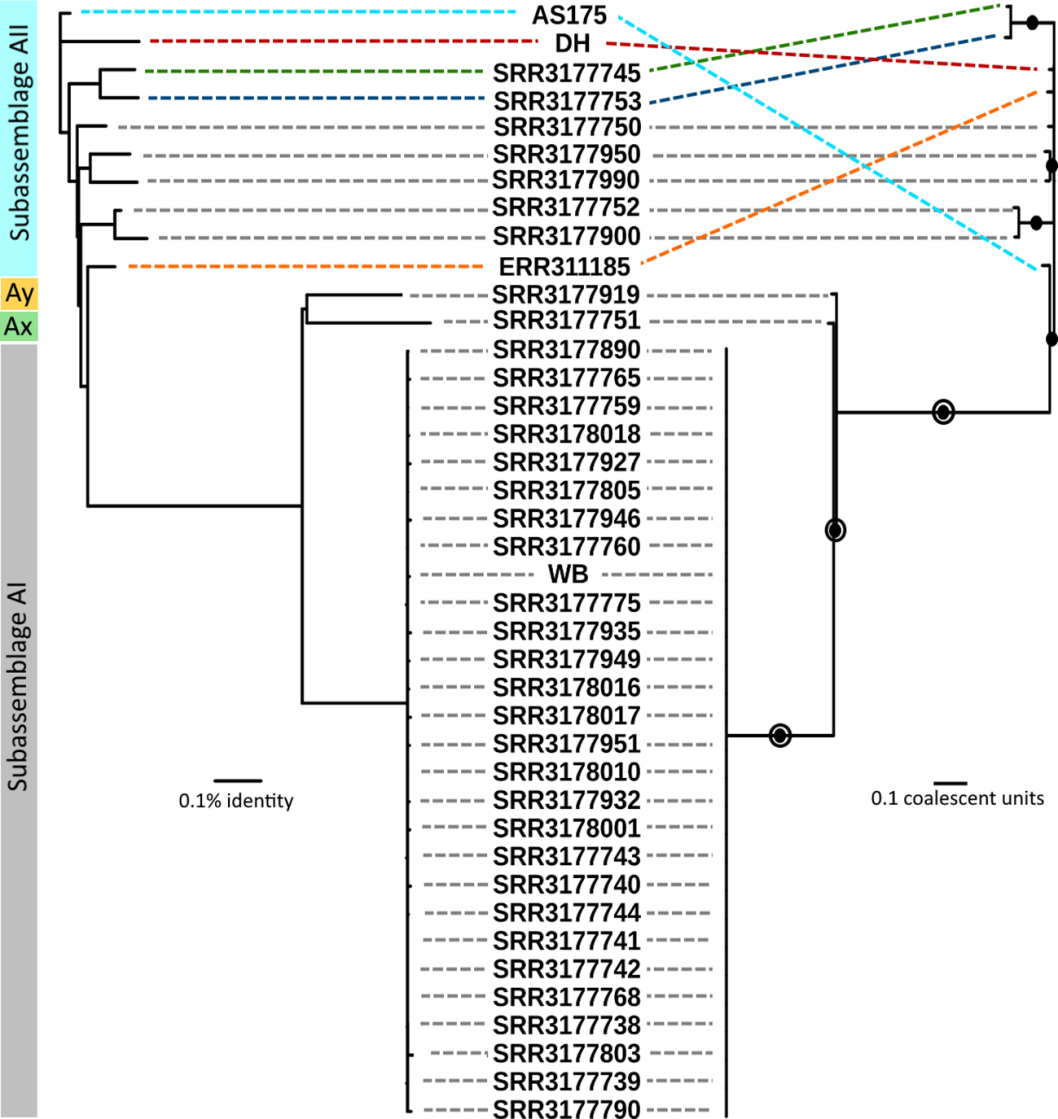
Comparison of genomic relatedness using ANI and multilocus coalescent estimations. Left side: ANI tree constructed from pairwise comparisons of draft genomes. Right side: Coalescent tree estimated by ASTRAL-III using gene trees from all pangenome loci. Filled circles indicate nodes with greater than 95% bootstrap support. Open circles indicate nodes with greater than 95% support in the set of trees estimated by STACEY. Grey dashed lines indicate concordant placement of a genome on opposing trees. Colored dashed lines indicate specific genomes which had differing placement.

Phylogenetic relationships between sub-assemblages within assemblage A are thought to be strongly conserved based on numerous studies employing multilocus sequence typing (MLST) and have been confirmed by comparative analyses of genomes (Monis et al., 1999; Sprong et al., 2009; Adam et al., 2013; Tsui et al., 2018; Ankarklev et al., 2015, 2018; Nash et al., 1985; Sulaiman et al., 2003; Read et al., 2004). Thus, the aims of our phylogenetic analysis were to quantify patterns of clonality for estimation of species boundaries. Genomic relatedness based on ANI is widely used to compare prokaryote genomes and was recently shown to be strongly correlated to, and thus interchangeable with, ML-based relatedness in *Giardia* across short evolutionary distances (Seabolt et al., 2021). Amongst all 41 genomes, pairwise ANI values ranged from 98.112% – 99.996% identity. Maximal clique enumeration (MCE), a graph-based clustering technique, using the same three loci and their percent identity cutoffs that were previously used with genomes of assemblage B (Seabolt et al., 2021), identified 11 clonal complexes within assemblage A: one large complex composed of all 29 sub-assemblage AI genomes and 10 complexes composed of 1 or 2 genomes each from sub-assemblages AII, Ax, and Ay (**Figure S1**). The Ax and Ay lineages both arise distinctly from all other genomes using both phylogeny estimation methods. The ANI tree confirmed that all genomes assigned to sub-assemblage AII arise from the same node, however the relationships between these genomes were more variable and did not show the same strongly clonal patterns as AI genomes (**Figure 2**, left side). Coalescent-based analysis using astral-III, a summary-based method to produce a species tree from a set of gene trees, estimated phylogeny with very concordant topology to the ANI tree, recovering well-supported nodes that corresponded to sub-assemblage AI, AII, and again recovered the Ax and Ay genomes as distinct lineages. The relationship of sub-assemblage AII genomes to one another is generally concordant with ANI as well, reflected by the recovery of several strongly supported (> 95%) subtrees in both cases. The agreement between the inferred topologies from both methods is illustrated in **Figure 2**. The MCE graph also identified two of these subtrees as clonal complexes of size 2 (SRR3177900 and SRR3177752; SRR3177745 and SRR3177753, shown in **Figure S1**). MCE analysis did not support the subtree pairing SRR3177950 and SRR317990, which may reflect differences between the character-based coalescent analysis and the distance-based ANI comparison across short evolutionary time scales.

We calculated within-lineage nucleotide diversity (π) along with raw and net divergence (D _xy_ and D_a_, respectively) for the AI and AII clades and for the global set of genomes using 133,160 SNPs annotated as silent by snpEff. The Ax and Ay genomes were included in the global set of genomes, however these could not be analyzed individually here since both lineages are represented by a single genome. Our estimates of π, D _xy_ , and D_a_ are summarized in **Table 1**. Within sub-assemblage AI, all three metrics are small (π = 0.01519 substitutions per site, D _xy_ = 1.467%, and D_a_ = 0.052%) and supported the existing understanding of AI as a strongly clonal population with average ANI > 99.9%. Genetic variation was approximately 10x higher across all three metrics within sub-assemblage AII than was found in sub-assemblage AI (π = 0.10033 substitutions per site, D _xy_ = 9.531%, and D_a_ = 1.059%). When we included all 41 genomes together as an all-vs-all “global” population, we estimated π = 0.25589 substitutions per site, D _xy_ = 24.965%, and D_a_ = 0.624%. Taken together, these results indicated substantial population structure within assemblage A, which we further investigated using Tajima’s D and Fu and Li’s D* and F* tests for neutral evolution (statistics and p-values in **Table 1**). These tests did not identify evidence of selection deviating from neutral expectations in sub-assemblage AI nor in the global population. However, all three tests showed significant evidence of non-neutral selection in sub-assemblage AII (Tajima’s D = -2.61, p < 0.001; D* = -5.11, p = 0.02; F* = -5.07, p = 0.02). Negative values of Tajima’s D can be interpreted as evidence for population expansion, which in this case is corroborated by the Fu and Li tests that are sensitive to population expansions or contractions across short evolutionary time scales (Tajima, 1989b; Aris-Brosou and Excoffier, 1996; Ramirez-Soriano et al., 2008). This potential expansion can be visually observed on the ANI tree in **Figure 2** for sub-assemblage AII – the long external branches and short internal branches yield a star-like topology from this node indicating a rapid expansion of clonal complex diversity.

**Table 1:** Average Divergence and Estimates of Nucleotide Diversity within lineages (across silent sites only).

Putative Group	No. Genomes	No. Polymorphic Sites (S)	π	D_XY_	D_a_	Tajima's D	D *p*-value	Fu and Li's D*	D* *p*-value	Fu and Li's F*	F* *p*-value
AI	29	9,624	0.01519	0.01467	0.00052	0.2538	0.10	0.23980	0.1	0.27518	0.1
AII	10	37,910	0.10033	0.09531	0.01059	-2.61011	< 0.001	-5.11782	0.02	-5.05721	0.02
Global	41	133,160	0.25589	0.24965	0.00624	0.34209	0.10	-0.06791	0.1	0.09810	0.1

These are the publsihed names of the test statistic used, e.g. F* and D*.

Statistical species delimitation with stacey and speciesDA supported speciation events at all four nodes corresponding to each of the four sub-assemblage clusters with a minimum of 96.9% (8721 of 9000 sampled trees) probability across each of the collapse heights tested (1e-3 to 1e-5). The remaining sampled trees all identified three species clusters with unstable topologies. In each case, the Ax or Ay cluster was merged with a different cluster, however the clusters corresponding to sub-assemblage AI and AII never appeared together in the same cluster for any gene tree (**Table S2**).

### Gene content diversity is substantial and stable between likely cryptic species

**Figure 3:**
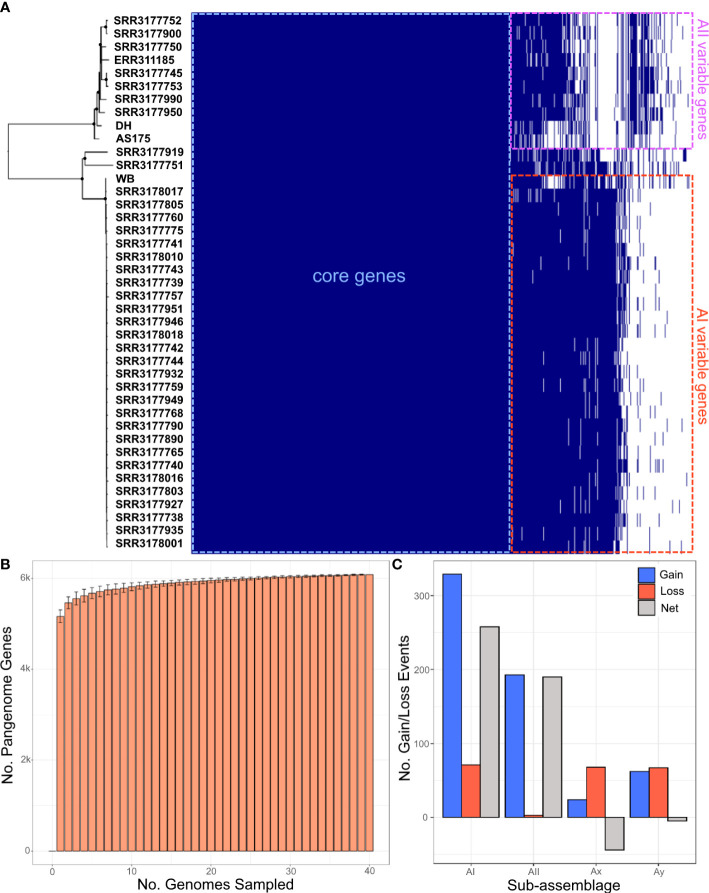
Characterization of the assemblage A pangenome. (**A**, left side) Neighbor-joining tree estimated from SNP distances between genomes. Filled circles indicates nodes with ≥ 95% bootstrap support. (**A**, right side) phyletic matrix of the assemblage A pangenome. Cells shaded in dark blue indicate a gene is present, and white-shaded cells indicate gene absence. **(B)** Rarefaction curve estimated from the phyletic matrix shown in **(A)** with 100 permutations. **(C)** Dynamics of gene gain/loss between sub-assemblages estimated by GLOOME. Blue bars indicate gene gain events, red bars indicate gene loss, and gray bars indicate net (gain – loss) change.

In addition to the core genes that were used for statistical species delimitation with stacey and speciesDA, we further explored how the variable gene content define species relationships by enumerating the assemblage A pangenome. The pangenome contained a total of 6,955 gene clusters, with 67% classified as core Assemblage A genes (n=4619), 24% classified as accessory genes (n=1694), and 9% of genes unique to one genome (n=642). On average, 5750 ± 266 genes were identified per genome analyzed (**Figure 3A**). We estimated the openness of the pangenome by rarefaction with 100 permutations. The rarefaction curve consistently leveled out, suggesting that the pangenome was fully (or mostly) sampled from these 41 genomes (**Figure 3B**). The Heaps law α parameter measures the rate of novel information discovery, with values of α > 1.0 indicative of a closed pangenome. Here, α was estimated to be 1.563, concordant with the rarefaction results. Neighbor-joining analysis of the gene presence/absence matrix resulted in a tree with robust support for the four sub-assemblages and showed strong agreement with all the other phylogenies based on ANI, coalescent, and SNP data (**Figure S2**).

We analyzed evolutionary dynamics of the assemblage A pangenome using the Gain/Loss mapping engine (gloome, Cohen et al., 2010) to further characterize functional differences between sub-assemblages. gloome computed probabilities for each potential gain or loss event along each branch of the ANI tree provided as reference, and assigned gene gains to the most probable branch, provided that the minimum probability of the gene being present on that branch is ≥ 95%. The results tallied a net gain of +258 and +190 genes in sub-assemblages AI and AII respectively, and a net loss of -44 and -5 genes for Ax and Ay (**Figure 3C**). We considered the overall net gain of gene content as evidence of adaptive evolution in AI and AII; however, we hesitate to consider the small net loss of genes in Ax and Ay as potential reductive evolution given the sample size of one genome each. The potential origin of genes identified as acquisitions were further analyzed using the orthologous group assignments based on protein sequence homology searches with eggNOG. We identified 129 of the gene acquisitions as variable surface proteins (VSPs), and 42 genes annotated as belonging to the Ankyrin repeat protein family, which are perhaps the result of gene duplications. Additionally, possible inter-domain horizontal gene transfer events appeared to be rare, with only 13 genes of likely bacterial origin found. Retrotransposon mobile elements accounted for an additional 33 acquired genes. All other acquired genes were annotated as eukaryotic genes from orthologous groups associated with the cell membrane and signaling (KOG categories T, U, and Z), metabolism (KOGs O, M, E, H), or with unknown function (KOG S).

### Unique gene content reveals adaptive functions tuned to host preference

Comparisons of unique gene content between sub-assemblages, host, and clonal complex groups (genome clusters sharing ≥ 99.9% ANI) identified amounts of gene content associated with surface proteins, signaling, cytoskeletal elements, or undetermined functions (KOG categories S,T, and Z; 38% – 100% among sub-assemblages, 37% – 100% among 5 hosts, and 18% – 50% among 9 clonal complexes). We did not identify any unique genes in genomes derived from sheep hosts, and one unique gene each in clonal complex A4 and A9 was identified. **Table S3** summarizes the proportion of unique gene content per group analyzed. We identified 42, 46, 11, and 5 variable surface proteins (VSPs) unique to sub-assemblage AI, AII, Ax, and Ay, respectively. Among hosts, 62 (12%), 28 (37%), 11 (100%), and 2 (2%) of unique genes were classified as VSPs for human, water, cat, and beaver sources. Finally, among clonal complexes, we found 52 (18%), 2 (3%), 2 (4%), 13 (35%), 6 (55%), and 6 (4%) VSP genes in clonal complexes A1, A3, A6, A7, A8, and A10 respectively (**Table S3**). Correlations between group size and the number of unique VSP genes identified significant association between unique VSPs per host (*r^2^* = 0.881, p = 0.0205, df = 4) and per clonal complex (*r^2^* = 0.9649, p < 0.0001, df = 9). However, we found non-significant association between the number of unique VSPs and sub-assemblage (*r^2^* = 0.755, p = 0.2446, df = 2).

**Figure 4:**
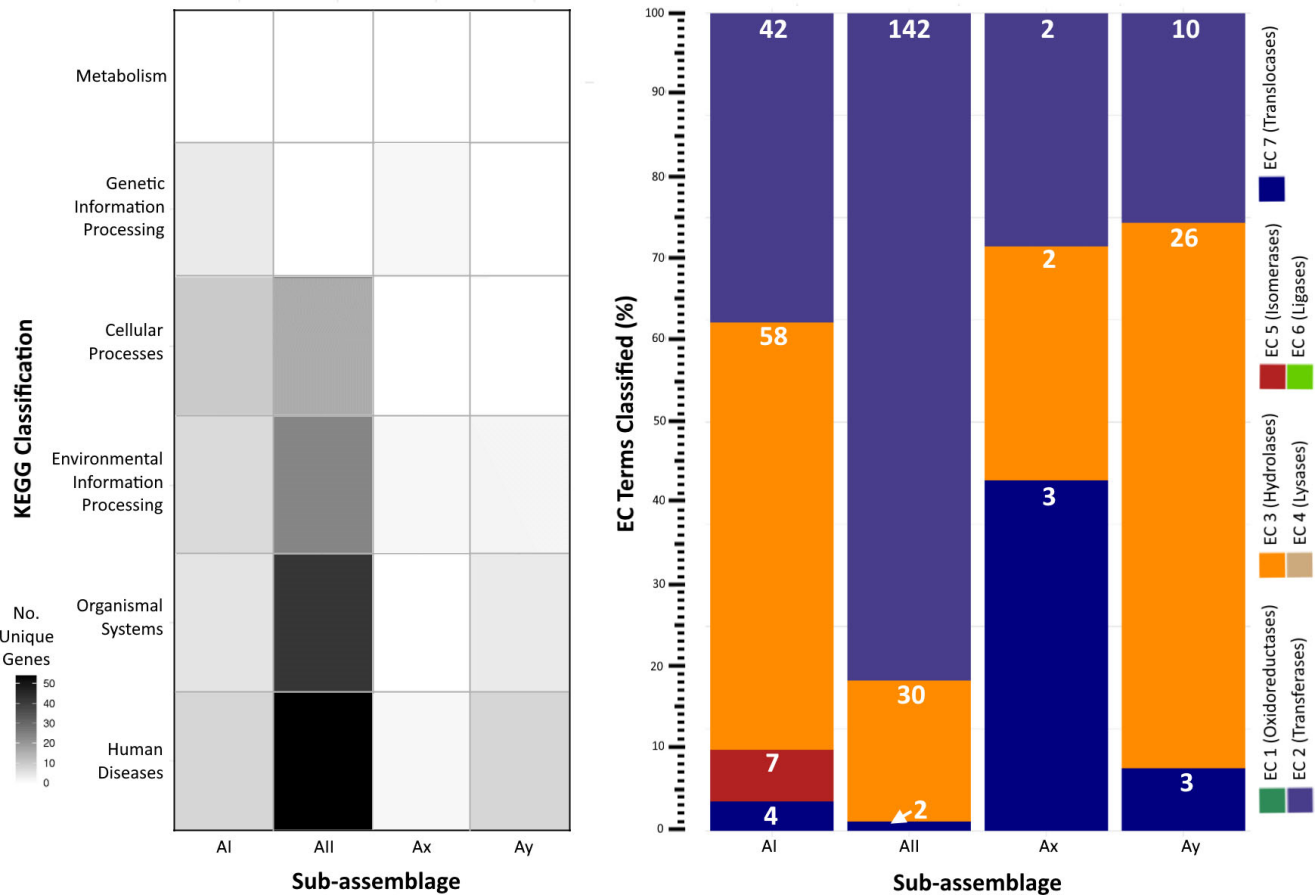
Functional pathway annotations of unique genes per sub-assemblage. Left: Heatmap of unique genes assigned to KEGG categories. Darker shading indicates greater number of unique gene content annotated to a given category. Right: Stacked bar chart of the percent distribution of EC terms applied to genes unique to a given sub-assemblage (some genes have multiple associated KEGG and/or EC terms, enumerated individually here). White numbers on each bar segment reflect the frequency count of each EC category per sub-assemblage.

KEGG and enzyme nomenclature (EC) terms (Kanehisa et al., 2016) assigned to unique genes per sub-assemblage further revealed adaptations primarily classified into the categories of Human Diseases, Organismal Systems, and Environmental Information Processing and similar distributions of genes annotated as hydrolases, transferases, and translocases (**Figure 4**). KEGG terms for sub-assemblage AII mostly align with Human Disease-associated pathways involved in host sensing and invasion, particularly the MAPK (K04371) signaling pathway utilized by other human parasites (e.g. *Toxoplasma*, *Leishmania*, *Trypanosoma*) to evade the host’s immune response. Gene content (n=39 genes with DIAMOND blastp e-values < 1e-20) mapping to this pathway was only identified in sub-assemblage AII genomes derived from human cases. 1 KEGG term (K01404) matching the GP63 gene, a surface antigen involved in host cell reception in the Leishmaniasis pathway, was discovered in the Ay genome but not in AII or any other sub-assemblage. No genes associated with known parasitic infection pathways were identified in sub-assemblages AI or Ax. In many cases, we were either unable to reliably assign KEGG terms to the gene *via* homology search or the predicted function was denoted as poorly characterized in the KEGG database (n = 118, 50, 8, and 28 genes for AI, AII, Ax, and Ay respectively). A full list of annotated genes and accompanying designations can be found in **Table S4**.

### Population dynamics rejects frequent panmixia and biogeography as major sources of genetic diversity between sub-assemblages

Based on the previous results indicating adaptive evolution and population structuring, we characterized potential population dynamics at finer resolution using pairwise comparisons between sub-assemblages. Wright’s *F _st_* is the proportion of genetic variance encapsulated in a sub-population (S) within the total population (T) (Wright, 1943; Wright, 1965; Weir and Cockerham, 1984). Here, our total population was all 41 genomes, and we defined “subpopulations” as the sub-assemblages for our calculations. *F _st_* values ranged from 0.78259 to 0.90029 and was lowest between sub-assemblage AII and Ax and highest between AI and Ay (**Table 2**). P-values for all pairs except for Ax vs. Ay were significant (p < 0.0001). *F _st_* could not be calculated between Ax and Ay since there was only one genome each. High estimates of *F _st_* (> 0.20) can be interpreted as evidence of population structuring when genetic mutations approach fixation within subpopulations being compared. D _xy_ and D_a_ are estimates of raw and net per-site nucleotide substitutions. Across silent sites, D _xy_ ranged from 32.44% to 57.9%, and D_a_ ranged from 30.78% to 52.63% (**Table 2**). Our hierarchical AMOVA considered biogeographical region (USA (18% of genomes), Canada (70%), Europe (5%), New Zealand (7%)) as a potential source of variation within sub-assemblages, which may have explained some of the previous results in support of population structuring. The AMOVA results rejected this hypothesis, finding that 96.9% of all variation was found between sub-assemblages (86.5% of total variance, df=3, φ = 0.865, p = 0.0001) and within sub-assemblages (10.4% of total variance, df=30, φ = 0.896, p = 0.0001), but not between regions within sub-assemblages (3.25% total variance, df=2, φ = 0.241, p = 0.1648) or between individual genomes within regions (1.15% of total variance, df=5, φ = -0.015, p = 0.3856). AMOVA results are further described in **Table 3**. Small negative values for variance sometimes occur due to higher variability within groups than between groups and can be functionally considered as zero in these cases. The reported φ statistic is analogous to Wright’s *F _st_ *. AMOVA also indicated significance among individual genomes within sub-assemblages, which can be explained as structuring within sub-assemblages (particularly sub-assemblage AII), which we have described as clonal complexes.

**Table 2:** Pairwise estimate of genetic diversity and divergence between lineages across silent sites.

Group 1	Group 2	No. Group 1 Genomes	No. Group 2 Genomes	π_total_	D_a_	D_xy_	*F_ST_*	*F_ST_ p*-value
AII	Ax	10	1	0.19531	0.49988	0.55731	0.78259	< 0.0001
AII	Ay	10	1	0.19631	0.50537	0.56281	0.78569	< 0.0001
AII	AI	10	29	0.23661	0.49219	0.55939	0.89858	< 0.0001
AI	Ax	29	1	0.03952	0.30947	0.31924	0.89352	< 0.0001
AI	Ay	29	1	0.04082	0.32907	0.33884	0.90029	< 0.0001
Ax	Ay	1	1	0.31946	0.31946	0.31946	NaN	NA

NaN, not a number; NA, not applicable.

**Table 3:** Analysis of Molecular Variance results from Assemblage/Region hierarchies.

	AMOVA Results			Components of Covariance			
Variance Component	DF	Sum Sq	Mean Sq	Variance	% Total Variance	*p*-value	φ-statistics
Between assemblages	3	5177.164	1725.721	274.340	86.516	0.0001	0.86520
Between Region within Assemblage	2	151.151	75.576	10.296	3.247	0.1648	0.24086
Between Genomes within Region	5	155.382	31.076	-0.473	-1.150	0.3856	-0.01456
Within Assemblage	30	987.636	32.921	32.981	10.383	0.0001	0.89618
Total variations	40	6471.333	161.783	317.084	100.000	--	--

## Discussion

The public health burden and zoonotic potential of *G. duodenalis* underscore the importance of revising *Giardia’*s taxonomy, particularly using whole-genome sequences as they become available, which will help advance our understanding of the parasite’s evolution and biology such as virulence factors and host preferences, improve source tracking of outbreaks, and assess the public health risks posed by zoonotic strains. Molecular typing studies have illustrated important epidemiologic differences between sub-assemblages AI and AII, revealing that AI is strongly clonal across its global distribution and is more often identified in animals than in human cases, while AII exhibits a larger breadth of genetic diversity and is the most common assemblage A sequence type identified in humans (Xiao and Fayer, 2008; Sprong et al., 2009; Ryan and Cacciò 2013; Thompson and Ash, 2016; Ankarklev et al., 2018). Thus, recognizing the sub-assemblages as unique species, following a blueprint laid out in the proposal by Thompson and Monis (2011) for the higher assemblages, has the potential to be very impactful for public health investigations and prevention strategies. The body of evidence examined in the present study revealed stable and distinct genomic signatures characterizing these sub-assemblage A clades, and we believe compelling biological and epidemiological justification exists to elevate sub-assemblage AII to species rank and propose the name *Giardia hominis* for this taxon. We discuss this and the broader question of taxonomic revisions to *Giardia* in detail below. In addition, the bioinformatics framework described in our study could be applied to resolve relationships between additional microbial eukaryote taxa for which taxonomic revisions are problematic due to cryptic species or species complexes.

Challenges to species differentiation are common in protozoan taxa owing to the scarcity of morphological characters that can be used to distinguish many closely related microbial species. Further, the capability of molecular methods to distinguish these species can be limited by the availability of suitable primers to amplify divergent homologous genes separated by large evolutionary distances, which can result in poor phylogenetic resolving power. The genomic data analyzed in this study show clear evidence of phylogenetic signal suggesting that, although the evolutionary split between AI and AII is likely recent and their genomes remain very similar at the sequence level, key biological differences such as clade-specific gene content reveal that the genome of sub-assemblage AII is well adapted to humans as its primary host, consistent with previously known epidemiologic trends and biological factors such as improved axenic culture growth of AII isolates using human serum in growth media (Cacciò et al., 2008; Ankarklev et al., 2015). The close relationship between sub-assemblages AI and AII at -for example- the 18S SSU-rRNA gene is characterized by only a single T->C transition within the approximately 1400 bp length of the locus, which is not captured by the most common approx. 293 bp target that is often amplified using the *Giardia*-specific PCR assay published in Hopkins et al. (1997) and modified in Applebee et al. (2003). Likewise, only 6 and 7 SNPs dispersed across the entire length of the SSU-rRNA gene differentiate assemblage E from sub-assemblage AI and AII respectively, despite assemblage E sharing 84% genome aggregate ANI with both assemblage AI and AII (Jerlström-Hultqvist et al., 2010). Accepted ANI standards for prokaryotes use 95% ANI as a rule-of-thumb for distinguishing one species from another, thus 84% ANI would clearly identify assemblage E as a separate species from assemblage A. However, while ANI concepts are widely accepted for prokaryotes, ANI has seen comparatively very little use with microbial eukaryotes and thus no direct corollary for rule-of-thumb species boundaries exists to compare with the 95% ANI threshold. Nevertheless, such high sequence similarity at the 18S SSU-rRNA locus is reflective of the slow-evolving nature of ribosomal genes due to strong selective pressures and purifying selection. Our results suggest that the divergence between the sub-assemblages is sufficiently recent that insufficient time has elapsed for the ribosomal genes to accumulate enough mutations for a robust phylogenetic signal at this locus. This “insufficient time” hypothesis may also provide a broader explanation for why assemblage A was, and continues, to be regarded as one “species” despite the presence of at least three distinct sub-assemblage divisions that are easily distinguishable by commonly sequenced subtyping loci, namely *tpi*, *gdh*, and *bg*, or even by allozyme analysis (Monis et al., 1999; Sprong et al., 2009). In fact, the established MLST scheme using the three genes mentioned above to subtype assemblage A isolates is based primarily on diversity *within the sub-assemblages* rather than at the assemblage level and indeed, no phylogenetic divisions comparable to A’s sub-assemblage have been identified in any other assemblage in the *G. duodenalis* complex.

**Figure 5:**
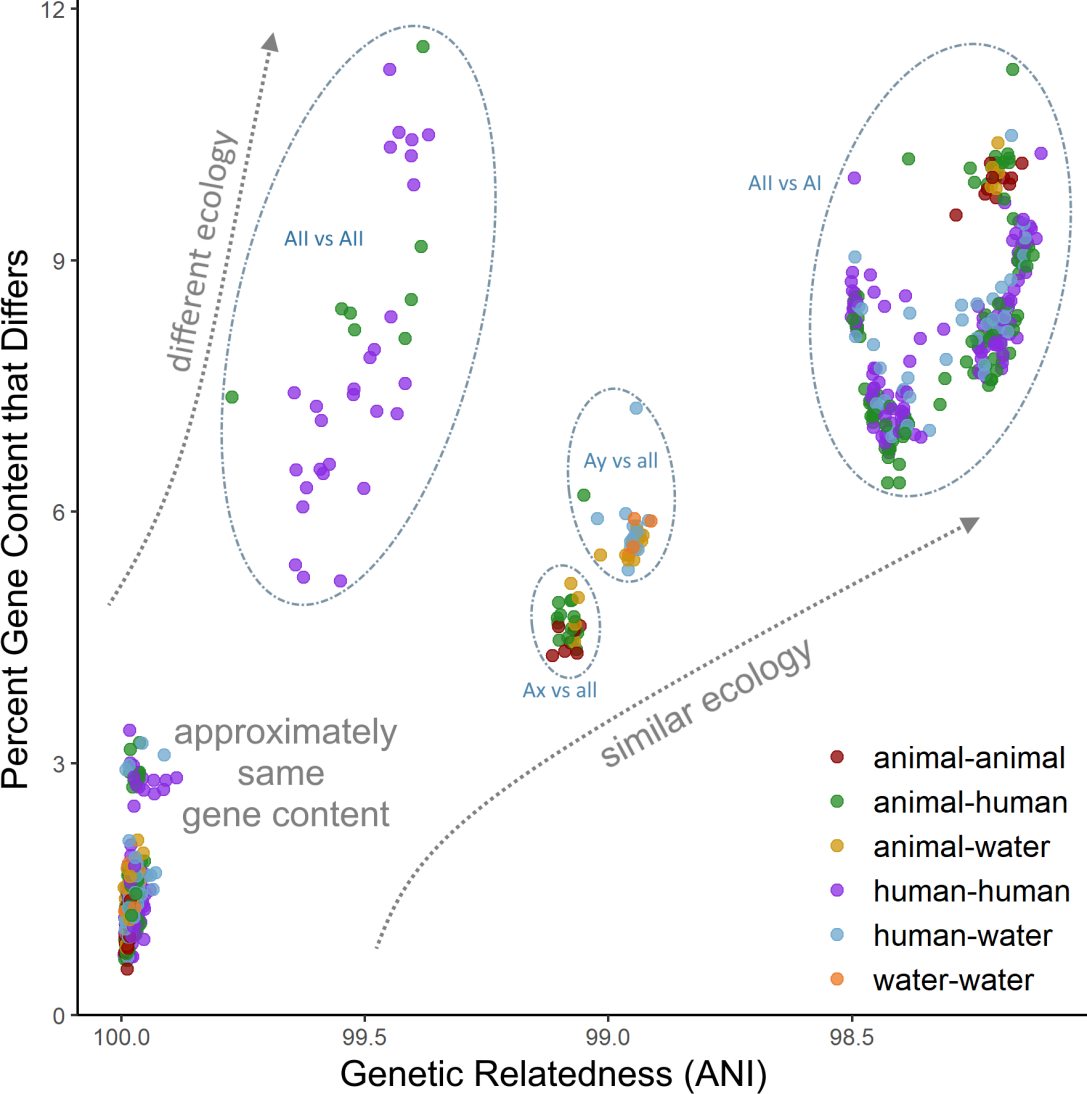
Comparison of gene content diversity with genomic relatedness. Each dot represents a pair of genomes. Dots are colored according to the host origin of the genomes.

Phylogenomic methods such as the coalescent and distance-based estimations conducted in our study were able to overcome phylogenetic resolution limitations of the common *tpi*-*gdh*-*bg* marker MLST scheme, and consistently identified clear distinctions between four sub-assemblage clades, including strong support for the uniqueness of both the Ax and Ay lineages. In addition, each sub-assemblage contains unique gene content that likely underlies key differences in function and ecology and provides an additional layer of resolution beyond simple sequence differences. Of particular interest are the VSPs, which make up a substantial portion of the assemblage A genome (up to 4%) and are thought to be related to differential virulence between isolate genotypes (Adam et al., 2010; Ankarklev et al., 2010; Xu et al., 2020). Our results indicate that these genes are likely associated with host preference as well as virulence, which may have implications for future public health investigations and merit further investigation into cross-transmissibility of potentially zoonotic strains. Nevertheless, the distribution of unique gene content and acquired biological functions provide evidence that the observed adaptive evolution is perhaps related to expansion of the ecological niche of the sub-assemblages and by extension, clinical relevance. Furthermore, our SNP-based population genetics analyses indicate patterns of both non-neutral evolution in sub-assemblage AII along with patterns of vertical inheritance in both the AI and AII clades, suggesting that the accumulated divergence between genomes is under selection pressure and is not solely random genetic drift. When considered together, the high degree of concordance among phylogeny estimation and statistical methods suggests that population structure and gene content are closely linked in these genomes, likely due to vertical inheritance within the clonal complexes (or more broadly, within sub-assemblages) driven by unique ecological niche preferences between the sub-assemblages rather than panmixia or recombination among the total population. We found that very closely related genomes (> 99% ANI) can differ by as much as 10-12% in their gene content (**Figure 5**), underscoring the conclusion that gene content differences are also important factors to consider when characterizing species boundaries among cryptic clades (Konstantinidis et al., 2006; Weigand et al., 2015). However, caution should be taken when interpreting these genome clusters, which may be biased towards strains that cause symptomatic infections in humans and thus may not be representative of the breadth of naturally occurring diversity.

Clonal complex enumeration using identical clustering thresholds identified in Seabolt et al. (2021) identified sub-assemblage AI as a single genetically homogenous complex but revealed sub-assemblage AII as a group of heterogenous complexes similar to the range of diversity of clonal complexes identified in assemblage B. Interestingly, unique gene content identified in the assemblage A sub-assemblages typically belong to the same functional categories as unique genes in assemblage B complexes (**Tables 3** and **S3**); however, we found no evidence for geographic factors explaining the diversity patterns as was the case with assemblage B. This signal of biogeographic endemism was instead replaced with evidence for host type being more significantly associated with specific complexes. These trends should be further scrutinized for differences that would be informative to epidemiologic investigation by sampling of additional genomes previously characterized broadly as assemblage A and comparing them using the novel MLST method described in Seabolt et al. (2021). Additional testing of this PCR technique described in in Seabolt et al. (2021) determined that it is suitable for assemblages A-E, and assemblage G. No DNA was available for testing of assemblage F, but the data suggest that this method can be expected to be widely applicable across assemblages in the *G. duodenalis* complex. To accompany wider use of this typing scheme, a suitable nomenclature using a revised species taxonomy should be developed to avoid confusion with the previous assemblage typing schemes commonly seen in literature (sub-assemblages AI, AII, AIII, BIII, BIV) and the common *tpi*-*gdh*-*bg* scheme (subtypes AII-1, AII-2, etc.).

### Comparisons to case studies of other microbial eukaryote genomes

Comparative data from a range of eukaryotic whole genomes revealed an intermediate “grey zone” of speciation (Roux et al., 2016), in which gene flow between diverging populations is limited and isolated populations transition to discrete species. This grey zone typically spans the range of 0.5% - 2.0% net synonymous divergence (D_a_) and appears to be robust in the absence of additional evolutionary evidence such as life history or ecology. Thus, this divergence threshold is useful as a framework for delineating cryptic microbial clades for which traditional species concepts or methods of estimating RI are not suitable (Roux et al., 2016; Singhal et al., 2018). The sub-assemblage clades examined in our study are shown to be substantially more divergent genome-wide than the 2.0% grey zone upper threshold (**Table 2**), which further supports our hypothesis that assemblage A, as currently understood, represents a multispecies complex of recently diverged cryptic species (sub-assemblages AI, AII, and AIII at minimum). Despite no representative genome of sub-assemblage AIII being available for analysis at the time of this writing, its phylogenetic relationship to AI and AII implies that it would also meet the same criteria for species elevation (Ryan and Cacciò, 2013), which remains to be evaluated empirically.

Similar considerations have been highlighted for other examples of clinically important microbial organisms such as (prokaryotic) botulinum-toxic *Clostridium* and non-pathogenic *Clostridium* strains (Weigand et al., 2015), or the protozoan parasites *Cryptosporidium cuniculus*, *C. hominis*, and *C. parvum* (Robinson et al., 2010; Nader et al., 2019), which are very closely related but have significant biological differences that merit species recognition. The genomes of *C. parvum* and *C. hominis* share 97% ANI and raw divergence (D _xy_ ) of 0.031 (Nader et al., 2019). *C. hominis* and *C. cuniculus* are even more closely related (99.06% ANI), substantially closer than any sub-assemblage AI-AII genome pair (98.31% ANI). Our results between sub-assemblages mirror the relationships observed between these *Cryptosporidium* species closely, which are hypothesized to be strongly conserved due to recent speciation events, like our conclusions for assemblage A clades. Notably, the most salient argument for recognition of both *C. hominis* and *C. cuniculus* was host preference for humans and lagomorphs, respectively, (Robinson et al., 2010; Morgan-Ryan et al., 2002). Likewise, here, we also reason that sub-assemblage AII’s preference for human hosts is a compelling justification for species recognition. The subsequent use of whole-genome sequences to characterize the biological signatures of closely related *Cryptosporidium* genomes has supported the argument of host preference to differentiate *C. cuniculus* and *C. hominis* and the *C. parvum* subspecies *C. p. anthroponosum* (Feng et al., 2017; Nader et al., 2019; Xu et al., 2019). Sequencing of additional *Giardia* genomes are needed to make similar comparisons possible in the future, particularly as new methods are developed to facilitate sequencing of *Giardia* specimens which are rare, historical, and/or cannot be propagated in culture.

### Broader implications for *Giardia* taxonomy

Despite common acknowledgement that *G. duodenalis* is a species complex, no comprehensive taxonomic revision has materialized yet due to historical uncertainty about the identity of existing type material, which have not been preserved using methods compatible with DNA analysis to determine the correct identity of morphologically indistinguishable species (approximately 40 species of *Giardia* described prior to ca. 1990; Cacciò et al., 2008; Thompson and Monis, 2011). In the case of *G. duodenalis* assemblages A and B, which are understood to have broad host ranges, attempts to anecdotally recognize the identity of the originally described isolates based on the host, type locality, or original descriptions have not reached broad consensus (Thompson and Monis, 2011). Thus, we propose that the most effective model going forward is to proceed with a systematic revision of *G. duodenalis* taxonomy, designating genomically-characterized neotype specimens when applying existing names to redescribed taxa (in accordance with ICZN Article 75) and sequenced genomes accompanying any future species classifications and new type material (ICZN Article 72.5). The proposal for a revised taxonomy of *G. duodenalis* put forward by Thompson and Monis (2011) attaches the name *G. duodenalis* to assemblage A, specifically to a rabbit-derived specimen, which would be unlikely to be a sub-assemblage AII representative and almost certainly not sub-assemblage AIII. Therefore, if the name *G. duodenalis* remains attached to sub-assemblage AI, then sub-assemblages AII and AIII could be assigned new names going forward (as well as the Ax and Ay lineages, should additional sampling demonstrate that these are also recognizable as unique species). Newly recognized species of *Giardia* are conventionally named based on their typical host, which in the case of sub-assemblage AII is challenging because the oldest available name for a human-derived isolate of *Giardia* is *G. enterica*, which is proposed to be the correct name for assemblage B (Thompson and Monis, 2011; Monis et al., 2009). The only other names described from a human-derived *Giardia* specimens are *G. lamblia* and *G. intestinalis*, which are both already often encountered in literature (although use of these names is incorrect – they are synonyms of *G. duodenalis* based on existing species circumscriptions). To avoid confusion with either of these existing names, we propose the name *Giardia hominis* for sub-assemblage AII, in light of multiple lines of evidence that humans are the preferred natural hosts of this species.

Order Diplomonadida Wenyon, 1926
Family Hexamitidae Kent, 1881
Genus *Giardia* Künstler, 1882
***Giardia hominis* n. sp.**

Type host: Homo sapiens (human).

*Type locality*: West Virginia, USA

*Type material*: ATCC PRA-246 (strain designation “DH” or “D. Hall”), collected from the duodenum of a symptomatic human patient (Nash et al., 1985).

*Representative genomic DNA sequences*: Whole-genome contig sequences are available for the primary type strain DH under the GenBank accession no. AHGT00000000 (Adam et al., 2013). Contigs of additional representative isolates (“AS175” and “AS98”) are available under the accession no. GCA_001493575.1 and GCA_900069105.1 (Ankarklev et al., 2015).

*Etymology*: The name *Giardia hominis* is chosen to reflect this organism’s natural preferred host (*Homo sapiens*).

*Remarks:* This species is morphologically indistinguishable from other assemblages of *G. duodenalis*, having to oval to elliptical-shaped cysts (avg. 10-14 µm in length/6-8 µm in width) with four nuclei, and pyriform trophozoites with “claw-shaped” or “ball-peen hammer”-shaped median bodies and two nuclei (avg 12-15 µm in length/6-8 µm in width). Morphological characters should not be relied upon for robust species identification, which should be deferred whenever possible to DNA sequence analysis of whole genomes or high-resolution loci such as those described in Seabolt et al. (2021). The “DH” isolate was selected as the primary type specimen since it is well established in cultivation and widely available to researchers as a model isolate. Molecular detections of *G. hominis* have been occasionally reported in cats, dogs, cattle, sheep, goats, pigs, and non-human primates using the *tpi-gdh-bg* typing method, establishing the zoonotic potential of this species.

## Conclusions

This study presents statistical and biological evidence that we believe qualifies the sub-assemblages of assemblage A for elevation to species rank alongside the other genetic assemblages of *Giardia duodenalis*. Genome sequences of representatives of assemblages AI, AII, B, C, D, and E are now available (Morrison et al., 2007; Jerlström-Hultqvist et al., 2010; Adam et al., 2013; Ankarklev et al., 2015; Wielinga et al., 2015; Kooyman et al., 2019), but assemblages AIII, F, G, and H have not been characterized genomically yet. However, the lack of genome sequences for the latter assemblages should not prevent taxonomic revision of well-sampled lineages to better reflect and advance the widely acknowledged view that *G. duodenalis* is a species complex. We recommend against the use of sub-species ranking to avoid confusion with any existing intra-species or subtyping nomenclatures and because biological justification for subspecies, traditionally based on observed phenotypic plasticity, is unconvincing in *Giardia*’s case. Additionally, we propose that recognition of new species must be accompanied by sequenced genomes, which have the discriminatory power to resolve cryptic lineages.

It remains unclear from the results of our study how to consider the Ax and Ay genomes, since they represent unique lineages under our framework but do not correspond to known, well-sampled groups from which broader conclusions related to breadth of their ecological niche can be drawn. Each of these lineages should be expanded on by further sampling of genomes from environmental and wildlife strains, and analysis of their gene content, which is likely to provide key insights into unique ecological niches and novel public health understanding.

We acknowledge the overall small sample size as a key limitation to the broader conclusions of this work, which are also likely biased towards symptomatic human infections. Increasing the number of genomes per sub-assemblage, particularly sampled from environmental and wildlife strains, would allow for improvement in the overall estimates of genetic diversity, population structuring, and relationships among lineages. We were not able to include outgroup genomes in phylogenomic analyses since the nearest available genome is assemblage E, which shares (only) 84% identity with assemblage A and thus was too evolutionarily distant for statistical species delimitation among closely related lineages. Likewise, in population genetic analyses, no outgroup was used in within-lineage comparisons for the same reasons, which may lead to overestimating statistics for the tests of neutrality and supporting hypothesis about recent population expansion. However, it is unlikely that these limitations affected the main conclusions of our study since results were consistent across methods and our methods are characterized by different assumptions or requirements. Overall, our results further elucidate connections between genetic relatedness, gene content, and ecology and suggest that this relationship could be of high importance to advancing our understanding of *Giardia’*s epidemiology and highlight the necessity of increasing whole-genome sequencing for this group.

## Data availability statement

The datasets presented in this study can be found in online repositories. The names of the repository/repositories and accession number(s) can be found in the article/**Supplementary Material**.

## Author contributions

Study design: MS, KK, and DR. Technical analyses: MS. Manuscript preparation: MS, KK, and DR. All authors contributed to the article and approved the submitted version.

## Funding

This work was partly funded by the U.S. National Science Foundation, award number 1759831 (to KK). The findings and conclusions in this report are those of the authors and do not necessarily represent the official position of the Centers for Disease Control and Prevention.

## Conflict of interest

Author MS was employed by Leidos Inc.

The remaining authors declare that the research was conducted in the absence of any commercial or financial relationships that could be construed as a potential conflict of interest.

## Publisher’s note

All claims expressed in this article are solely those of the authors and do not necessarily represent those of their affiliated organizations, or those of the publisher, the editors and the reviewers. Any product that may be evaluated in this article, or claim that may be made by its manufacturer, is not guaranteed or endorsed by the publisher.
