# Supplementary figures and images for: Genomic comparisons confirm Giardia duodenalis sub-assemblage AII as a unique species
Source: Front Cell Infect Microbiol. 2022 Oct 17;12:1010244. doi: 10.3389/fcimb.2022.1010244 (PMC9618722; doi:10.3389/fcimb.2022.1010244)

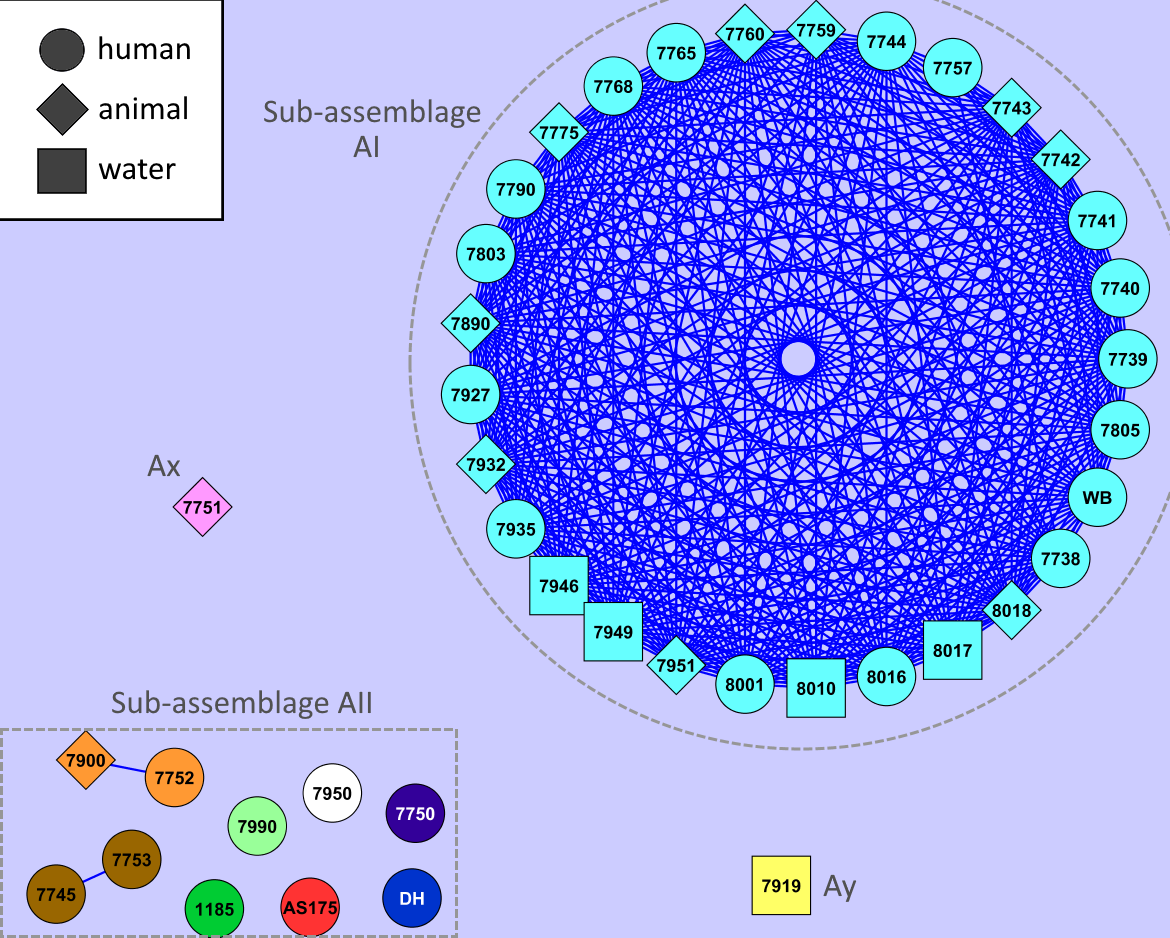

Supplement: Supplementary file 1 [file Image_1.tiff]

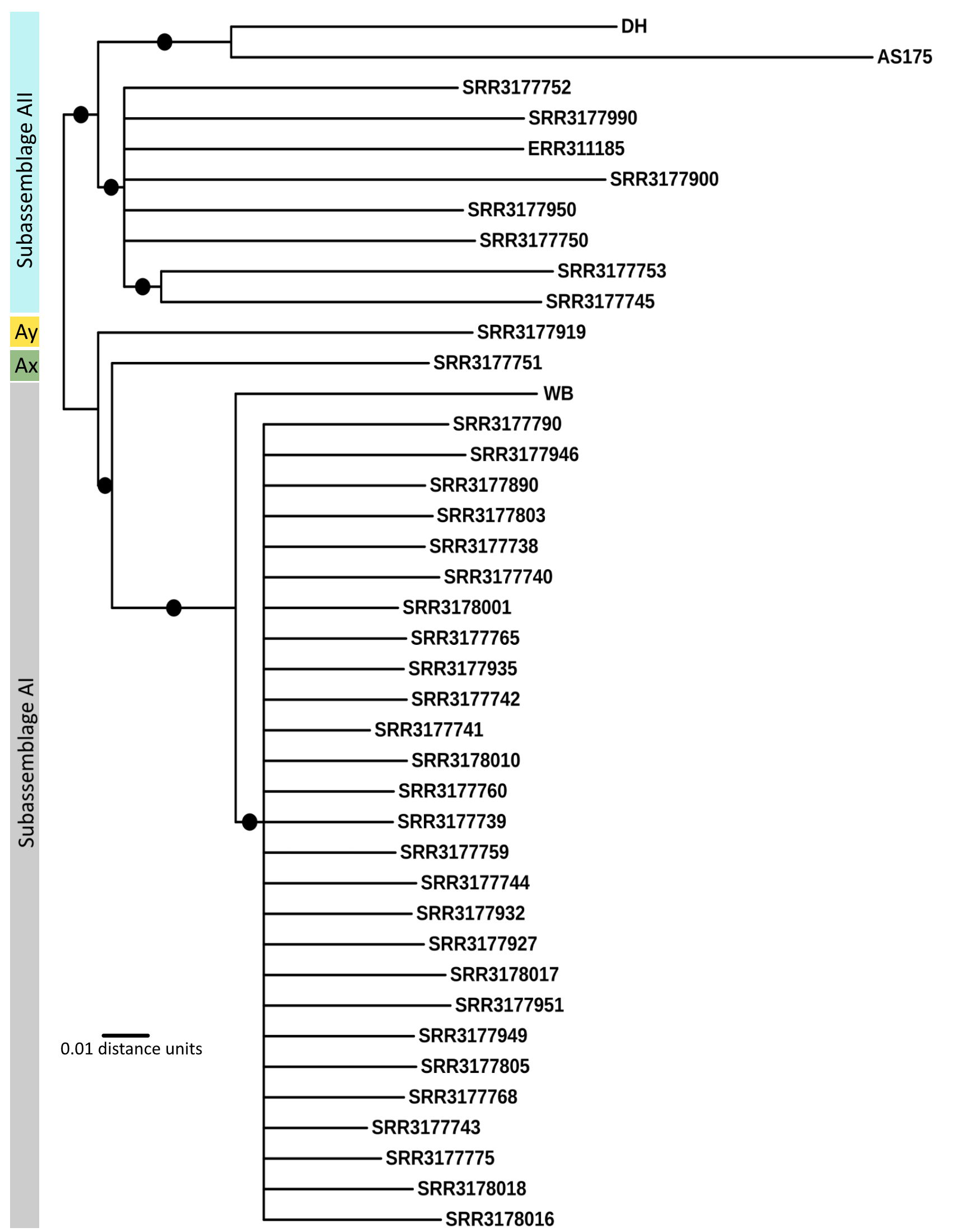

Supplement: Supplementary file 2 [file Image_2.tiff]
